# Gyroscope Technology and Applications: A Review in the Industrial Perspective

**DOI:** 10.3390/s17102284

**Published:** 2017-10-07

**Authors:** Vittorio M. N. Passaro, Antonello Cuccovillo, Lorenzo Vaiani, Martino De Carlo, Carlo Edoardo Campanella

**Affiliations:** 1 Photonics Research Group, Dipartimento di Ingegneria Elettrica e dell’Informazione, Politecnico di Bari, via E. Orabona n. 4, 70125 Bari, Italy; martinodecarlo@gmail.com (M.D.C.); ce.campanella@qopsys.com (C.E.C.); 2 QOpSyS SRL, Via Matteotti 23, Gioia del Colle, 70023 Bari, Italy; an.cuccovillo@gmail.com (A.C.); l.vaiani@qopsys.com (L.V.)

**Keywords:** mechanical gyroscopes, optical gyroscopes, MEMS gyroscopes

## Abstract

This paper is an overview of current gyroscopes and their roles based on their applications. The considered gyroscopes include mechanical gyroscopes and optical gyroscopes at macro- and micro-scale. Particularly, gyroscope technologies commercially available, such as Mechanical Gyroscopes, silicon MEMS Gyroscopes, Ring Laser Gyroscopes (RLGs) and Fiber-Optic Gyroscopes (FOGs), are discussed. The main features of these gyroscopes and their technologies are linked to their performance.

## 1. Introduction

The term “gyroscope”, conventionally referred to the mechanical class of gyroscopes, derives from the Ancient Greek language, being the Physics of the “precession motion”, a phenomenon also observed in ancient Greek society [1].

**Figure 1:**
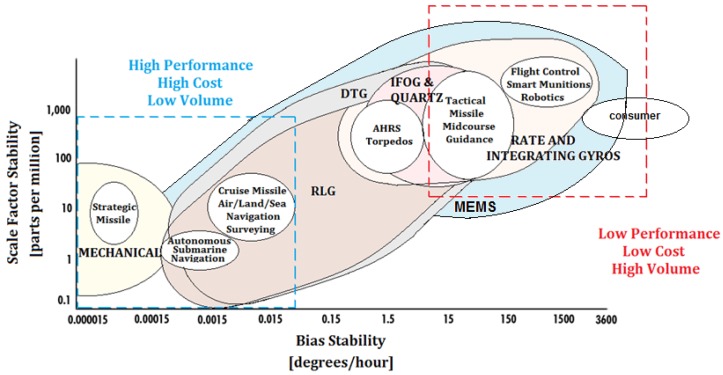
Scale factor stability (i.e., the accuracy of the gyroscope in monitoring the sensed angular velocity), expressed in parts per million (ppm), as a function of the bias stability (intrinsically dependent on the gyroscope technology) for Mechanical Gyroscopes, Ring Laser Gyroscopes (RLG), Interferometric Fiber-Optic gyroscopes (IFOG), Quartz, Dynamically Tuned Gyroscopes (DTG), Rate and Integrating Gyroscopes and MEMS.

Gyroscopes are devices mounted on a frame and able to sense an angular velocity if the frame is rotating. Many classes of gyroscopes exist, depending on the operating physical principle and the involved technology. Gyroscopes can be used alone or included in more complex systems, such as Gyrocompass [2], Inertial Measurement Unit [3], Inertial Navigation System [4] and Attitude Heading Reference System [5]. In this paper, a review of the more commercially diffused classes of gyroscopes is presented. In particular, mechanical gyroscopes (Section 2); optical gyroscopes (Section 3), including Fiber Optic Gyroscopes (FOGs) [6,7,8] and Ring Laser Gyroscopes (RLG) [9,10]; and Micro-electromechanical system (MEMS) gyroscopes [11,12] (Section 4) have been considered by focusing attention on the operating principles and different improvements in commercial architectures in terms of performance. For all classes of gyroscopes, being angular velocity sensors, the major issues are related to the errors in measuring the angular velocity. For this reason, one of the more important merit figures is the stability of the scale-factor. Scale factor represents the sensitivity of the optical gyroscope, while the accuracy of the gyroscopes, which is inversely proportional to the sensitivity and takes into account the measurement errors due to the noise, can be expressed through the resolution, *R*, or, in the RLGs, by the Angle Random Walk (*ARW*), linking *R* with the bandwidth, *B*, of the measurement system through *ARW* = *R*/[60*sqrt*(*B*)] [13]. A minimum scale-factor stability leads to small sensor errors and requires better instruments and improved accuracy, bringing higher cost of the system. Thus, gyroscope performance and costs are directly related to the application requirements. For more details about performance and applications, see Figure 1.

Since the 19th century, mechanical gyroscopes (see the left-bottom spectrum in Figure 1), classified as displacement gyroscopes and rate gyroscopes, are the historical ones consisting in a toroid-shaped rotor that rotates around its axis while, since the 20th century, optical gyroscopes (see the spectrum in the middle of Figure 1) operate by sensing the difference in propagation time between counter-propagating laser beams traveling in opposite directions in closed or open optical path. The main diffused types of optical gyroscopes are IFOG and RLG, which both exploit the physics of the Sagnac effect. For applications requiring very high performance, the ring laser gyroscope is currently more diffused and has the bigger market share.

Micro-Electro-Mechanical System (MEMS) gyroscopes are motion sensors that detect and measure the angular motion of an object. They measure the rate of rotation of an object around a particular axis: 1-axis, 2-axis, and 3-axis. Although initially used for expensive military applications, now they are also adopted for low cost commercial applications (low performance at the top-right end of the spectrum in Figure 1) of consumer electronics for Automotive, Defense, Industrial and Medical applications. The increased demand for mobile devices is also responsible for the growth of the MEMS gyroscopes market. The cost of MEMS gyroscopes is expected to reduce drastically in the next years, leading to an increment in the use of these devices.

The MEMS and optical gyroscopes, in particular Interferometric Fiber-Optic gyroscopes (IFOG), are replacing many of the current systems using Ring Laser Gyros (RLGs) and mechanical gyroscopes. However, among the optical gyroscopes, applications requiring extremely high scale factor stability continue to be achieved only with RLG. In Figure 1, the scale factor stability (i.e., the accuracy of the gyroscope in monitoring the sensed angular velocity), expressed in parts per million (ppm), as function of the bias stability (a parameter that is intrinsically dependent from the gyroscope technology), is reported. Depending on the scale stability, an extensive range of applications is considered with reference to the gyroscope technology (i.e., Mechanical, RLG, IFOG, Quartz, Dynamically Tuned Gyroscopes (DTG), Rate and Integrating Gyroscopes, and MEMS), and the performance in terms of Scale Factor Stability vs. Bias Stability.

Better performance, such as the highest costs and the smallest production volume, are related to the gyroscope technologies placed at the left-bottom corner while the lowest performance, such as the lowest costs and the highest production volume, are placed at the right-top corner of Figure 1, including the consumer electronics applications. Among the gyroscopes technology in this review, we cover the more diffused and commercial gyroscope technologies (i.e., Mechanical, RLG, IFOG and MEMS) with the relative applications reported in the following figure.

## 2. Mechanical Gyroscopes

A mechanical gyroscope essentially consists of a spinning mass that rotates around its axis. In particular, when the mass is rotating on its axis, it tends to remain parallel to itself and to oppose any attempt to change its orientation. This mechanism was invented in 1852 by physicist Léon Foucault during his studies of the Earth’s rotation. If a gyroscope is installed on gimbals that allow the mass to navigate freely in the three directions of space, its spinning axis will remain oriented in the same direction, even if it changes direction.

A mechanical gyroscope shows a number of physical phenomena, including precession and nutation. In the following sections, the main operating principles of the mechanical gyroscopes are reported, with reference to the Inertial Navigation Systems.

### 2.1. Principle of Mechanical Gyroscopes: Gyroscopic Effects

The basic effect upon which a gyroscope relies is that an isolated spinning mass tends to keep its angular position with respect to an inertial reference frame, and, when a constant external torque (respectively, a constant angular speed) is applied to the mass, its rotation axis undergoes a precession motion at a constant angular speed (respectively, with a constant output torque), in a direction that is normal to the direction of the applied torque (respectively, to the constant angular speed) [14]. External forces acting on the center of mass of the rotating part do not affect the angular position of the rotation axis.

**Figure 2:**
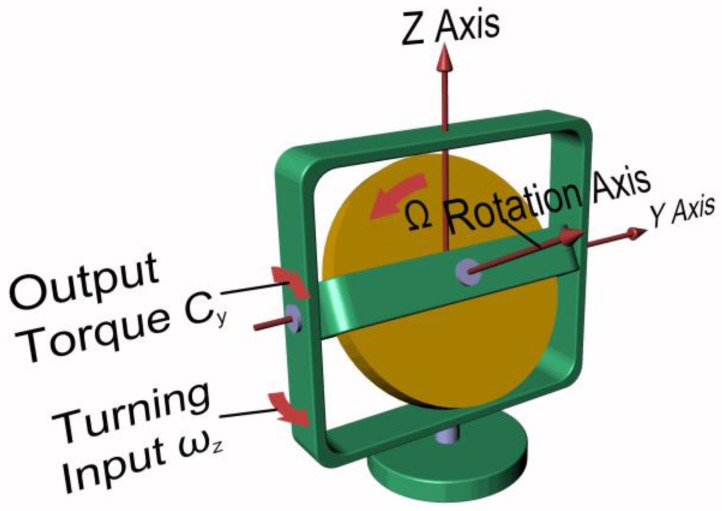
Elements of a mechanical gyroscope and main parameters.

Referring to Figure 2, the simplified equations governing the physical phenomenon are:

(1) $$C_{y}=-I\Omega \omega_{z}$$

(2) $$C_{z}=I\Omega \omega_{y}$$

where *C_y_* and *C_z_* are the torques acting along *y* and *z* axis, respectively; *I* is the polar mass moment of inertia of the spinning mass; *Ω* is the angular velocity of the spinning mass along the rotation axis; and *ω_y_* and *ω_z_* are the precession speeds along *y* and *z* axis, respectively. As shown by Equations (1) and (2), the output torque due to an imposed precession motion is proportional to the inertia and the rotational speed of the spinning mass.

### 2.2. Mechanical Displacement Gyroscopes

The primary application of gyroscopic effects consists in the measurement of the angular position of a moving vehicle. The spinning mass is mounted upon a gimbaled frame, allowing rotation along two perpendicular axes.

The gimbaled frame of the gyroscope is attached to the vehicle and it is free to rotate, while the rotation axis of the spinning mass keeps its angular position during the motion of the vehicle. The variation of the absolute angle of the vehicle can be simply associated to the relative variation of the angle between the rotation axis of the mass and a fixed direction on the frame of the gyroscope.

Another useful application of this physical effect is that exploited in gyrocompasses: when external torques are not applied to the frame, the gyrocompass keeps the angular position of a pointer to North direction, independently of the path followed by the vehicle. The advantage of such a mechanical system is that it is immune to magnetic fields that can cause deviations on the pointer angle.

### 2.3. Mechanical Rate Gyroscopes

**Figure 3:**
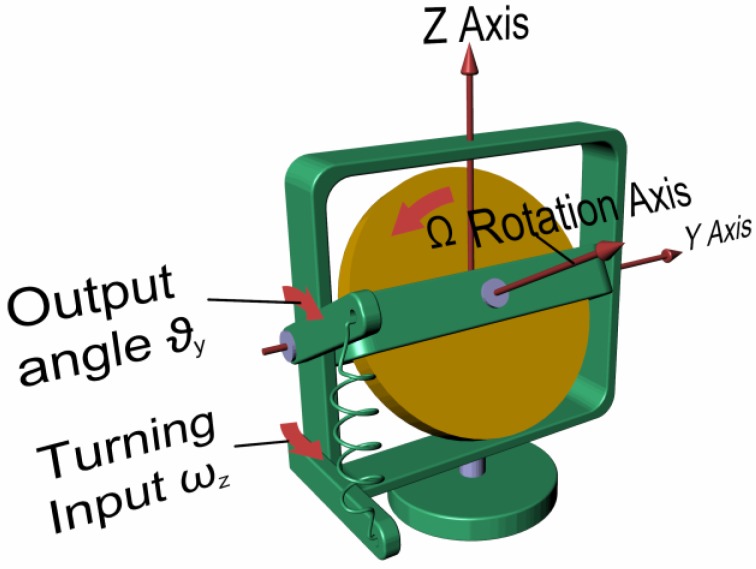
Mechanical rate gyroscope.

Rate gyros measure the angular speed of a vehicle during rotary motion. According to Equation (1), it is possible to notice that when an angular speed (e.g., *ω_z_*) is imposed to a gimbal and hence to the spinning mass, a proportional torque (e.g., *C_y_*) appears along a direction that is perpendicular to the direction of imposed angular speed, as shown in Figure 3.

If a spring system of known stiffness is opposing and balancing the output torque, it is possible to compute the imposed angular speed through the measurement of the output angle (e.g., *ϑ_y_*) assumed by the inner gimbal frame over time.

### 2.4. Description of Common Mechanical Gyroscopes

A mechanical gyroscope consists of:

(1) A spinning wheel mounted on two gimbals: This allows it to undergo precession motions along two perpendicular directions.
(2) A rigid frame with rotating bearings: The mechanical components in relative motion are subjected to friction, which in turn leads to measurement drifts over time. The main goal in gyroscope design is to build a frictionless and perfectly balanced device. To minimize friction, high-precision bearings and special lubricants are used or, in many critical applications, magnetic suspensions or fluid-suspended configurations [15];
(3) Sensing systems (pick-offs): These are capable of reading angular displacements between the two adjacent gimbals and to transduce them into electric signals by means of potentiometers, resolvers or encoders, thus constituting the input for a computing unit.

## 3. Optical Gyroscopes

Optical gyroscopes operate by sensing the difference in propagation time between counter-propagating beams travelling in opposite directions in closed or open optical paths. A rotation-induced change in the path lengths generates a phase difference between the counter-propagating light beams. This rotation-induced phase difference physically consists in the Sagnac effect, being the basic operating principle of all optical gyroscopes.

**Figure 4:**
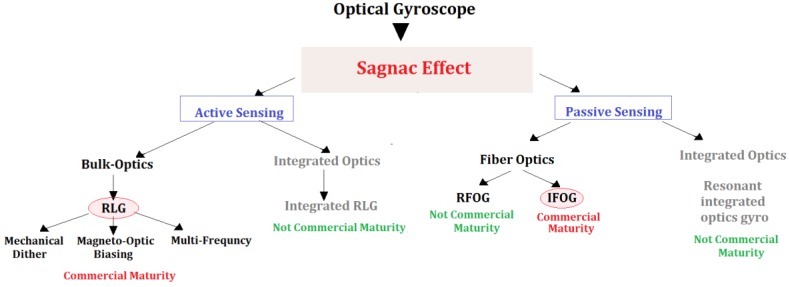
Classes of optical gyroscopes.

Based on the measurement technique of the Sagnac effect, it is possible to classify the optical gyroscopes. The two main different typologies of optical gyroscopes consist in active and passive architectures (see Figure 4). In the active configurations, the closed-loop optical path (i.e., the ring cavity) contains the optical source, forming a ring laser. The active configurations can be built in Bulk Optics or in Integrated Optics technology, although only the Bulk Optics solutions have achieved commercial maturity.

Among the Ring laser gyros, there are different categories depending on the method employed to overcome the lock-in effect (i.e., a condition for which the active gyroscope response results insensitive to low rotation rates) which occurs at low rotational rates (tens of degrees/hour). Lock-in can be reduced by introducing a mechanical dither, a magneto-optic biasing, or by using of multiple optic frequencies configuration. Differently, in passive architectures, the optical source is external to the closed optical loop (i.e., a fiber coil) as in the Interferometric Fiber Optic Gyroscope. Ring Laser Gyroscopes and Interferometric Fiber Optic Gyroscopes, whose features differ in terms of size, weight, power requirements, performance, and cost, are the more diffused optical gyroscope technology [13].

### 3.1. Sagnac Effect

The underlying operating principle of almost all optical gyroscopes is the Sagnac effect. It was discovered in 1913 by George Marc Sagnac as a result of the study of the dynamics of Earth rotation by Michelson-Morley [16], and observation of electromagnetic wave in non-inertial reference systems [17]. Sagnac principle, as derivation of the general relativity theory, states that two counter-propagating optical beams propagating in a ring structure change their relative phase if the ring is rotating; thus, it is possible to relate the phase change to the angular speed of the ring.

As example, by considering an open circular light path of few centimeters and a rotational speed of one revolution per second, according to Sagnac effect, the optical beams path difference is of 0.00000025 mm, which is too small for angular velocity detection.

In 1966, the scientist E.O. Schulz-Du Bois, who was working at the embodiment of a ring laser based angular velocity sensor, proposed a ring laser gyroscope shaped as a hollow cylindrical mirror, in which light described a circular path. To make visible the rotation phenomenon in the ring cavity, two optical beams at the same wavelength, but counter-rotating, were introduced. The two optical beams reciprocally interfered and produced a permanent wave, stationary with respect to an external inertial reference system. The stationary wave is composed by nodes and anti-nodes, whether the mirror is rotating or standing. A detector is mounted on the ring framework, solidly rotating with it, so that, during rotation, by counting nodes and anti-nodes of the standing wave through the detector, it is possible to evaluate the angular velocity of the ring.

Indeed, the number of nodes is two times the length of the ring divided by the light wavelength. Knowing the number of counted nodes in the time unit through the detector, it is possible to calculate the angular velocity of the ring. The scale factor can be expressed as a constant directly proportional to the ring perimeter and inversely proportional to the ring area. For more details about the mathematical model of the Sagnac effect, see Section 3.1.1.

In reality, mirrors used for ring laser gyroscopes are never perfect. The mirror imperfections cause light back-scattering and energy loss that give rise to a sort of interaction between the propagating electromagnetic (e.m.) waves and the moving medium. As result, the permanent wave is no more perfectly stationary with respect to inertial reference but drifts following the rotation of the ring. Generally speaking, there is no relative motion between mirrors and standing wave so that a false zero speed signal is detected. This effect is called lock-in (see Section 3.2.1).

#### 3.1.1. Sagnac Effect in Vacuum and in a Medium

As mentioned before, all the rotational optical sensors are based on Sagnac effect that generates a difference of optical path Δ*L* proportional to angular velocity *Ω* [6].

**Figure 5:**
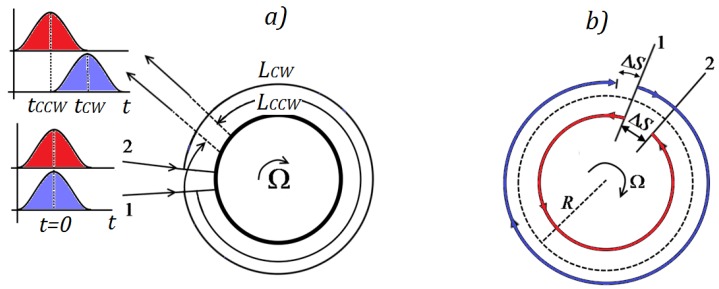
In a disk rotating with a clockwise (CW) angular velocity: (**a**) different rotation induced optical paths of the clockwise and counter-clockwise (CCW) optical beams, *L_CW_* and *L_CCW_*, respectively; and (**b**) identical difference, Δ*S*, between the rotation induced optical paths of the clockwise and counter-clockwise optical beam, and the standing optical path.

We consider a disk with radius *R* that rotates over a rotational axis perpendicular to the plane of the disk, with an angular speed *Ω*; the difference of optical path Δ*L*, due to light propagation in opposite directions along the perimeter, is given by:

(3) $$\Delta L=\frac{4A}{c_{0}}\Omega =2\Delta S$$

where *A* is the surface enclosed by the perimeter *L* and *c*_0_ is the speed of light in vacuum. The rotating disk is depicted in Figure 5: for a given point on perimeter, labeled 1, identical photons propagate in clockwise and counter-clockwise directions. If the initial angular velocity is null, photons that travel at speed of light in vacuum *c*_0_ will arrive at starting point 1 after a trip length of 2*πR* in time *t* = 2*πR*/*c*.

If the initial angular velocity is not null, photons that propagate in counter-clockwise direction, called CCW, will arrive at the starting point, now called 2, due to the motion caused by the rotation, after a trip length of L_CCW_, shorter than 2*πR*, given by:

(4) $$L_{CCW}=2\pi R-R\Omega t_{CCW}=c_{CCW}\cdot t_{CCW}$$

where *RΩ* is the tangential angular speed of the ring and *t_CCW_* is the time to cover the distance *L_CCW_* that is equal to the product of speed of light in counter-clockwise direction *c_CCW_* and time *t_CCW_*. In vacuum, it results *c_CCW_* = *c*_0_.

In the same way, the clockwise photons, called CW, will arrive at the starting point, now called 2, due to the motion caused by the rotation, after a trip length of *L_CW_*, longer than 2*πR*:

(5) $$L_{CW}=2\pi R-R\Omega t_{CW}=c_{CW}\;\cdot \;t_{CW}$$

The different sign of *RΩ* is related to the angular velocity sense: indeed, by supposing a clockwise rotation, clockwise photons cover a longer distance than counter-clockwise ones.

If light propagates in a medium characterized by a refractive index *n* [6], by considering the relativistic composition of propagation speed and tangential speed of medium, *c_CCW_* can be rewritten as:

(6) $$c_{CCW}\;=\frac{\frac{c_{0}}{n}+R\Omega }{1+\frac{R\Omega }{nc_{0}{}^{2}}}=\frac{c_{0}}{n}+R\Omega \left(1-\frac{1}{n^{2}}\right)+\ldots$$

While *c_CW_* is:

(7) $$c_{CW}\;=\frac{\frac{c_{0}}{n}-R\Omega }{1-\frac{R\Omega }{nc_{0}{}^{2}}}=\frac{c_{0}}{n}-R\Omega \left(1-\frac{1}{n^{2}}\right)+\ldots$$

where the right side of the previous expressions are the Taylor series expanded at the first term.

Now, Δ*τ* is equal to:

(8) $$\Delta t=t_{CW}-t_{CCW}=2\pi R\left[\frac{2R\Omega -(c_{CW}-c_{CCW})}{c_{CW}\cdot c_{CCW}}\right]$$

Substituting Equations (6) and (7) into Equation (8) we obtain:

(9) $$\Delta t=t_{CW}-t_{CCW}=2\pi R\left[\frac{2R\Omega -2R\Omega \left(1-\frac{1}{n^{2}}\right)}{\frac{c_{0}}{n^{2}}}\right]=\frac{2\pi R\;2R\Omega }{c_{0}{}^{2}}=\frac{4A\Omega }{c_{0}{}^{2}}$$

Δ*τ* results to be the same of that in vacuum case, and to this time difference correspond a phase shift of

(10) $$\Delta \Phi =\frac{2\pi \Delta t}{\frac{\lambda_{0}}{c_{0}}}=\frac{2\pi \Delta t}{\frac{\lambda }{c}}=\frac{8\pi A}{\lambda_{0}c_{0}}\Omega$$

where *λ* and *c* are wavelength and speed of light in medium. We can also rewrite Δ*L* as:

(11) $$\Delta L=\frac{\Delta \Phi }{2\pi }\lambda_{0}=\frac{4A}{c_{0}}\Omega$$

### 3.2. Ring Laser Gyroscopes (RLGs)

The ring laser gyroscopes (RLGs) are based on a ring laser (i.e., a annular cavity) where, due to the Sagnac effect, two independent counter-propagating resonant modes, intrinsically generated within the cavity through a gain medium, show of a frequency shift if the cavity undergoes a rotation [18]. The ring laser can be realized in bulk solid-state optics or in integrated optics, although the integrated optics solutions (e.g., semiconductor ring laser gyroscopes [19]) are not commercially mature.

**Figure 6:**
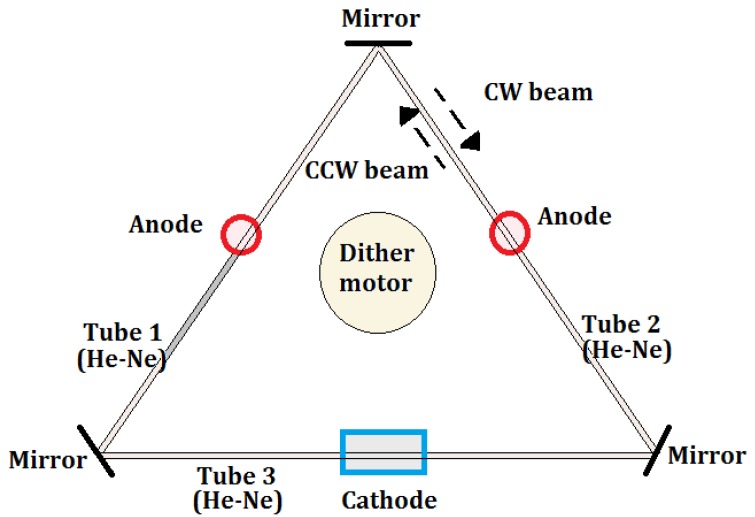
RLG made by a triangular optical resonator.

In one of the most diffused architectures, the RLG body is made from a triangular glass block (see Figure 6). Three air channels are drilled in the glass body and three mirrors are placed at each corner to create a triangular optical resonator. A low pressure He-Ne gas mix fills the three tubes. A high voltage electrical discharge is applied through the two anodes and the cathode for electrically pumping the optical cavity. Due to the action of the electrical pump, two independent counter-propagating laser beams (i.e., clockwise CW and counter-clockwise CCW), resonating at the same frequency, are generated inside the optical cavity. By making a partially reflecting mirror, it is possible to detect the angular velocity of the rotating system by reading the frequency change of the resonant behavior of the device or the interference pattern generated by the interaction of the CW and CCW laser beams, leading to a standing wave. The two read-out techniques are covered by the physics of the Sagnac effect. At very low rotation rates, the mirrors, being imperfect, produce backscattered light, which couples energy from a CW to a CCW. The backscattered light acts as a mechanism of frequency synchronization—lock-in of the two resonant beams at low rates of rotation.

#### 3.2.1. Lock-In Effect

The lock-in effect occurs only for conditions of weak mutual coupling between the two counter-propagating laser beams. Usually, this coupling is caused by different backscattering sources, depending on the adopted technology and architecture and it results in a response insensitiveness to low rotation rates. This operating region is called “dead band”. This backscattering-induced coupling between counter-propagating beams is also responsible of the creation of standing waves inside the resonant ring structure. These standing waves could generate an optically induced alternation of high and low index/absorption regions (i.e., an optically induced grating), which creates localized refractive index variations/losses, increasing the coupling and the lock-in. Possible solutions for circumventing this problem are represented by the dual mode ring laser gyroscopes [20,21], in which two modes oscillate simultaneously and interact to reduce the dead band, or by the insertion of a mechanical mechanism of oscillation around the sensor axis. This mechanism of oscillation, called dithering [22], is usually based on a complex mechanical system unable to completely eliminate the effect of the backscattering induced coupling. Moreover, sometimes the dithering vibrations cannot be applied to those ring laser gyroscopes mounted on platforms that are very sensitive to the vibrations. The other problem associated to ring laser gyroscopes, i.e., the mode competition, is due to the different gain-assisted photon-enhancement between the two counter-propagating resonant modes, whose eigenfrequencies are overlapped. By making different the two resonance frequencies of the two counter-propagating modes, it is possible to avoid the mode competition [23]. Integrated optics of ring laser gyroscopes solutions are yet not mature for commercial applications.

#### 3.2.2. Critical Parameters for RLGs

The critical parameters for ring laser gyroscopes are:

1. Size: Larger ring lasers gyroscope can measure lower rotation rates. The sensitivity of large ring laser gyroscopes increases quadratically with the size of the optical cavity.
2. Mirrors: The mirrors are fundamental elements for focusing and directing the laser beams to form the optical cavity.
3. Stability: The gyroscope body must be built within a substance that changes minimally in response to temperature fluctuations.
4. Gas: He-Ne generates beams with the most desirable features for large ring lasers. For gyros, in principle, any material that can be used to generate monochromatic light beams is applicable.

The RLGs covers the high-performance market. Size and weight of RLGs are other limiting factors, mainly due to the body and the mechanical dither assembly. Miniaturization of the RLG leads to a decrement in their reliability, reason for which integrated optics miniaturized solutions did not achieve a commercial diffusion. The power requirements of RLGs are high because power sources of several hundred volts at low currents are required.

#### 3.2.3. Recent Advances in RLGs

In the last ten years, important recent advances in RLGs have been achieved. To improve the performance of this kind of gyroscopes, different solutions have been proposed.

In 2007, Cai et al. [24] developed soft magnetic alloys that exhibited high strength to external stresses and to large temperature changes, allowing a wide temperature operating range for a four-mode ring laser gyro. They obtained a 0.01°/h zero excursion, with a 15-cm dimensioned differential laser gyro.

In 2008, Mignot et al. [25] reported for the first time the experimental achievement of a single-frequency ring-laser gyroscope, using a diode-pumped half-vertical-cavity semiconductor-emitting laser structure as a gain medium. The experimental setup had an overall perimeter of 50 cm. They obtained a scale factor of 716 Hz/(°/s).

In 2009, Schwartz et al. [26] could suppress nonlinear couplings induced by crystal diffusion and spatial inhomogeneities of the gain over a broad range of angular velocities in a solid-state ring laser gyro. The result was obtained by vibrating the gain crystal at 168 kHz and 0.4 µm along the cavity axis. The solid-state RLG used was made of a 22 cm long ring cavity, containing a 3 mm long diode-pumped Nd:YAG crystal. They showed that the level of angular random-walk noise in presence of mechanical dithering depends only on the quality of the cavity mirrors. The scale factor of the considered ring laser gyro was about 754 Hz/(°/s).

Large ring lasers can exceed the performance of navigational gyroscopes by several orders of magnitude. In [27], it is reported that an ultralarge ring He–Ne ring laser gyroscope (UG-2, 39.7 × 21 m^2^) has been built under ground. Earth rotation is sufficient to unlock it, with a Sagnac frequency of 2.18 kHz. The residual Sagnac frequency error, caused by backscatter coupling, is measured as <2 parts in 10^8^. The best stability achieved for an averaging time of about 2000 s. The scale factor is 7.67 × 10^5^ Hz/(°/s).

Direct dither control without external feedback was used in [28] in 2012 to avoid the lock-in effect for a ring laser gyro. A new design, that makes the system more compact and inexpensive, was proposed. Experiments showed that the accuracy using this method (ARW = 6.31 × 10^−4^°/√h and bias instability = 3.86 × 10^−4^°/√h) is nearly the same as that using the prior method with PZT as dither feedback.

While the most prevalent design of a RLG is the active gyroscope, in [29] a “passive” gyroscope is shown, in which the sensing cavity is tracked using external laser beams. This design is free from the deleterious lock-in effect observed in active systems and could be constructed using commercially available components. The core of the gyroscope was a squared free-space optical cavity of 75 cm side length. A sensitivity of about 5.7 × 10^−7^ (°/s)/√(Hz) above 500 mHz was achieved.

Recently, a modified expression for the Sagnac frequency of a large square ring laser gyro undergoing Earth rotation has been derived in [30]. The modifications include corrections for dispersion of the gain medium and the mirrors, for the Goos-Hanchen effect in the mirrors and for refractive index of the gas filling the cavity. The corrections were measured and calculated for the 16 m^2^ Grossring laser at the Geodetic Observatory Wettzell.

### 3.3. Interferometric Fiber Optic Gyroscopes (IFOGs)

The Interferometric Fiber Optic Gyroscopes (IFOGs) are based on an open optical path (i.e., a N turns fiber optics coil) where two independent counter-propagating modes, externally introduced through a laser sources, suffers of phase shift induced by the Sagnac effect, that is transduced in an interference pattern variation.

**Figure 7:**
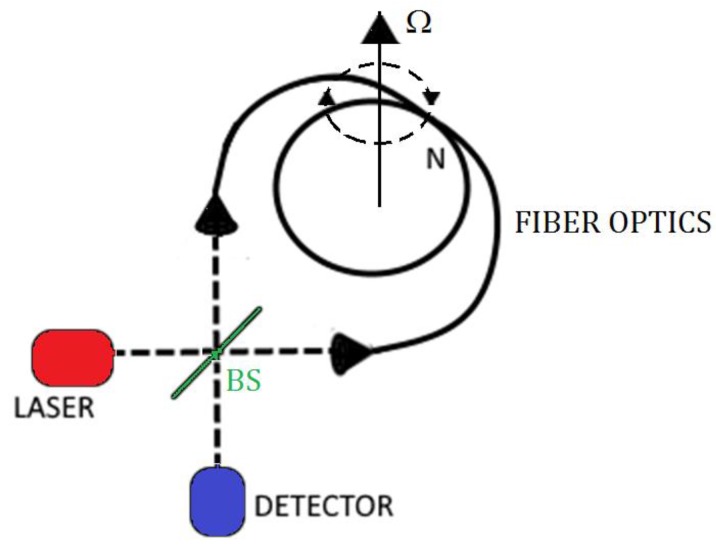
IFOG configuration.

A simple IFOG configuration is shown in Figure 7 where the light, which comes from a laser or appropriate light sources, is split by a beam splitter (BS) and then coupled at the coil ends of a single mode fiber. The light outgoing from coil is combined again in the beam splitter and, then, measured by a photo-detector. In absence of rotation, the two beams interfere constructively or destructively depending by the used beam splitter. If there is a rotation at an angular speed *Ω*, due to the Sagnac effect, the counter propagating beams will have a path difference Δ*L*, given by:

(12) $$\Delta L=\frac{\Delta \Phi }{2\pi }\lambda_{0}=\frac{4A}{c_{0}}\Omega$$

generating an interference fringe shift expressed as:

(13) $$\Delta z=\frac{LD}{\lambda_{0}c_{0}}\Omega$$

or alternatively a phase shift of:

(14) $$\Delta \Phi =\frac{2\pi LD}{\lambda_{0}c_{0}}\Omega$$

For a fixed length *L* of the sensor, as example obtained by fixing the coil diameter *D*, the sensitivity can be improved by increasing the total coil length *L* by adding a higher number of turns *N*, by taking into account that there is an upper limit to *L* due to the fiber attenuation.

**Figure 8:**
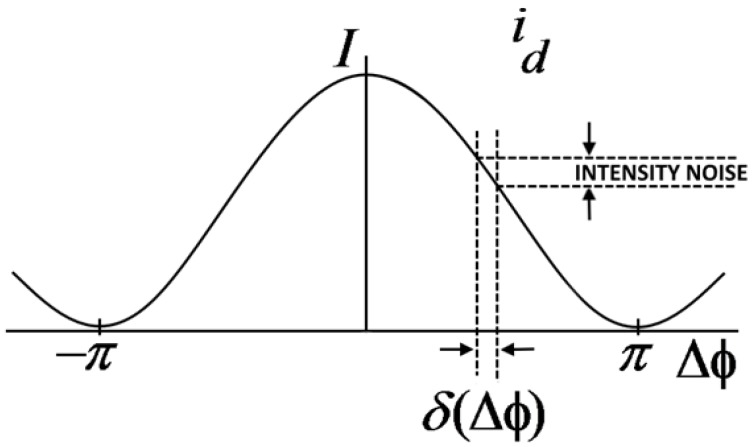
Intensity *I* of the output photo-current of the photo-detector.

The graph reported in Figure 8 shows the intensity of outgoing detector current as function of non-reciprocal phase shift Δ*Φ*. In this case, being *Ω* = 0, the intensity peak due to constructive interference is placed in Δ*Φ* = 0. In the case of rotation, Δ*Φ* slightly moves from zero position and the intensity of the detector current, *i_D_*, changes. The biggest change in the intensity of the detector current for an infinitesimal shift of Δ*Φ* appears at Δ*Φ* = ±*π*/2 where the slope achieves its maximum value.

Thus, applying a non-reciprocal bias of Δ*Φ* = ±*π*/2, we could fix a quiescent point in the maximum sensitivity region. Furthermore, in this way, an applied rotation causes a Δ*Φ* that generates a linear proportional variation of intensity, by allowing it to work in the linear region. An issue is related to the casual variations in light source intensity that cause perturbations in output current intensity. These perturbations are indistinguishable from variations of phase shift. However, it is possible to reduce or compensate fluctuations of source intensity reducing measurement uncertainty of *Ω*, produced by this phenomenon. Indeed, if the noise generated by the source is not taken into account, it is possible to consider the effect of shot noise that represents a random process.

In ideal conditions, indeed, measurement uncertainty of Δ*Φ* is limited by shot noise and is defined as follow:

(15) $$\partial \left(\Delta \Phi \right)=\frac{shot\;noise}{i_{D}\;slope}$$

Uncertainty results minimum when the slope of *i_D_* is maximum. This condition leads to:

(16) $$\partial \left(\Delta \Phi \right)=\frac{{\left(2ei_{D}B\right)}^{\frac{1}{2}}}{\frac{i_{D}}{\pi }}≅\frac{{\left(n_{PH}n_{D}\tau \right)}^{\frac{1}{2}}}{\frac{n_{PH}n_{D}\tau }{\pi }}$$

where *e* is the charge of electron, *n_PH_* is the number of photons per second impinging the photo-detector, *τ* is the mean time equal to 1/2*B* with *B* the measurement band and *n_D_* is the quantum efficiency of photo-detector.

The measurement uncertainty of *Ω* becomes:

(17) $$\partial \Omega =\frac{\lambda_{0}c_{0}}{2\pi LD}\partial \left(\Delta \Phi \right)$$

that, using Equation (14), becomes:

(18) $$\partial \Omega =\frac{\lambda_{0}c_{0}}{2\pi LD}\partial \left(\Delta \Phi \right)=\frac{c_{0}}{LD}\frac{\frac{\lambda_{0}}{2}}{{\left(n_{PH}n_{D}\tau \right)}^{\frac{1}{2}}}$$

### 3.4. Recent Advances in IFOGs and RMOGs

Different solutions have been proposed to improve Fiber Optic Gyroscopes performance in the last few years. In particular, a Resonant Fiber Optic Gyroscope (RFOG) uses dozens of meters of resonant fiber loops, thus reaching the same performance associated to kilometers of fiber coils into an I-FOG. The usage of Resonant Micro Optic Gyroscopes (RMOGs) allows obtaining good performance with smaller sizes, compared to FOGs. For all kinds of gyroscopes, bias drift is one of the most limiting factors.

The first experimental configuration of an IFOG was proposed in [31] by Vali and Shorthill in 1976. Very important improvements have been done since the earliest solutions to date. Next, some of the recent advances in Fiber Optic Gyroscopes (FOGs) and Resonant Micro Optic Gyroscopes (RMOGs) will be illustrated.

In 2006, the first air-core photonic-bandgap fiber gyroscope was reported [32]. Because the optical mode in the sensing coil largely travels through air (having low Kerr, Faraday and thermal constants than silica), a lower power and magnetic field dependences were obtained. With a 235 m fiber coil, a minimum detectable rotation rate of 2.7°/h and a long-term stability of 2°/h were achieved.

In 2007, a new open-loop configuration of an IFOG was presented. A single mode telecommunication optical fiber and an EDFA pumped with DFB laser were used as sensing coil and broadband source, respectively [33]. The Sagnac phase shift was extracted by a phase tracking circuit with an RC band pass filter, an amplifier and a modulator chip. It measured a bias stability of 1.57°/h and a sensitivity of 72 μV/(°/h). Moreover, a peak-to-peak noise of 8°/h was derived.

In 2009, a novel design of IFOG was presented by Yu et al. [34]. The proposed structure can reduce the effect of the polarization crosstalk and improves production efficiency. Through the application of all-digital closed-loop control and random modulation signal processing technology, the dead zone problem, typical of IFOG, caused by electronic cross-coupling, has been eliminated and the SNR of the gyro output has been improved. A zero-bias instability of 0.01°/h has been achieved and the random walk coefficient has reached 0.001°/√h.

In [35], it is shown that electro-optic polymers have been used in fabricating low loss phase modulators with low half-wave drive voltage for an Inertial Measurement Unit based on an IFOG. A novel technique was introduced for assessing the error caused by backscatter and an offset waveguide design was developed to suppress the interference of backscattered light. An average ARW of about 0.006°/√h and a bias uncertainty of about 0.02°/h were obtained.

In 2010, Yahalom et al. [36] presented a new IFOG. The design was based on an innovative approach that enabled the production of a small and low-cost gyro with excellent noise and bandwidth characteristics. The goal was to develop an inexpensive sensor in less than 50 cm^3^. The gyro was configured as a “split gyro”, where the light source, electronics and receiver are integrated in an external package and the sensor head was integrated in a robust and rigid package. The head sensor was 6.9 cm × 6.9 cm × 5 cm. They obtained a bias long-term stability of 0.2°/h, and an ARW of 0.0022°/√h.

In 2013, it was demonstrated that, by driving an IFOG with a laser of relatively broad linewidth (about 10 MHz), the noise would be reduced to 0.058°/√h, while the bias drift would be reduced to 1.1°/h [37]. Researchers used a 150 m fiber coil, with a 3.5 cm coil diameter. Using a laser instead of a broadband light source, it offers increased scale factor arising from the frequency stability of the laser. However, driving a FOG with a laser leads to large noise and drift, because of the coherent backscattering, the Kerr effect and polarization non-reciprocity. It was demonstrated that, carefully selecting the laser linewidth and using a symmetric phase modulation scheme, one can reduce these sources of error to a very low level.

In 2014, Wang et al. demonstrated a novel dual-polarization IFOG [38], which only needs one coupler and no polarizer. With a 2 km coil and an open-loop configuration, a bias instability of 0.02°/h and an ARW of 1.5 × 10^−3^°/√h were obtained.

The usage of RFOGs (Resonant Fiber Optic Gyroscopes) instead of IFOGs, allows to reduce fiber length, thus leading to lower dimensions. In the era of miniaturization, the possibility of integrating optical waveguides leads to even smaller solutions. Thus, RMOG is a promising candidate for applications requiring small, light and robust gyros.

In RMOGs, clockwise and counter-clockwise waves are phase-modulated at different frequencies to reduce backscattering induced noise. The effectiveness of this technique, however, is determined by the carrier suppression level. In the experiment reported in [39], carrier suppression is applied onto both the CW and the CCW waves at the same time to achieve higher total suppression. Ma et al. obtained a bias stability of 0.46°/s over 50 s with a silica waveguide ring resonator, having a ring length of 7.9 cm.

In 2013, a 2.5 cm diameter ring was equipped with a low-noise, low-delay digital signal processor based on FPGA [40], reaching a 0.67°/s bias stability over 3600 s. The proposed solution can detect a minimum rotation rate of 0.25°/s.

In the same year, Lei et al. [41] proposed a current modulation technique in an external cavity laser diode to construct a gyroscope system for the first time. The experimental results from the established RMOG setup demonstrate a bias stability of 2.7°/s (over 600 s) with a silica optical waveguide ring resonator having a ring length of 12.8 cm.

## 4. Micro-Electro-Mechanical System (MEMS) Gyroscopes

MEMS gyroscopes generally use a vibrating mechanical element as a sensing element for detecting the angular velocity. They do not have rotating parts that require bearings and this allows an easy miniaturization and the use of the manufacturing techniques typical of MEMS devices.

All MEMS gyroscopes with vibrating element are based on the transfer of energy between two vibration modes caused by the acceleration of Coriolis.

**Figure 9:**
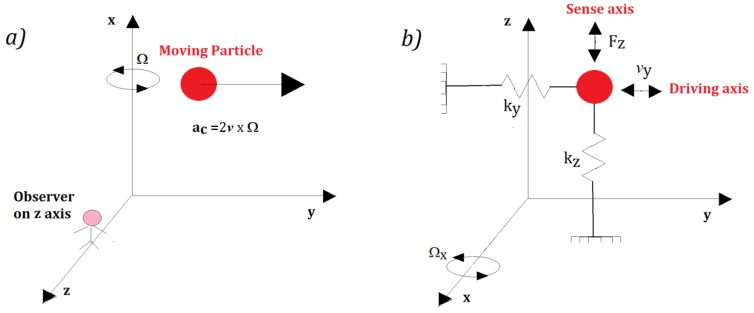
(**a**) Coriolis acceleration (a_c_) acting on a moving particle; and (**b**) mass-spring model of a MEMS gyroscope.

The Coriolis acceleration, proportional to the angular velocity, is an apparent acceleration that is observed in a rotating frame of reference. To better understand the concept, we can consider a particle of mass *m* moving in space with a velocity *v* (See Figure 9a).

Once fixed to the reference system in Figure 9a, if it is rotating with an angular velocity *Ω* = *Ω_x_i* (with *i* the unitary vector along the *x* axis) around the *x* axis, an observer, solidly anchored to the *z* axis, sees the particle moving along the *z* axis with a Coriolis acceleration equal to a_c_ = 2*v* × *Ω*, although a real force is not applied along the *z* axis. This is the key physical principle of the vibrating mass MEMS gyroscope, described like a mass-spring system (see Figure 9b).

The vibrating mass MEMS gyroscope has two orthogonal mechanical excitation modes along which the mass can move. If *k_y_* and *k_z_* are the elastic stiffness parameters proper of the frame, while *c_x_* and *c_y_* are the respective damping coefficients, the master equations result to be [42]:

(19) $$m\ddot{y}=-k_{y}y-c_{y}\dot{y}+F_{Drive}$$

(20) $$m\ddot{z}=-k_{z}z-c_{z}\dot{z}+F_{Cz}$$

(21) $$F_{z}=\left|2m\Omega \times v\right|$$

The primary mode is excited along *y* (drive axis) by applying a force *F_Drive_* (see Equation (19)), while the secondary mode along *x* (sense axis) is excited by the Coriolis force *F_z_* (see Equation (20)).

By knowing the mode drive axis mode, the displacement along the *z* axis is proportional only to the angular velocity *Ω* = *Ω_x_i*. Moreover, being *Q_y/z_* and *ω_y/z_* the quality factor and the resonance frequency of the driving mode (x) and the sensing mode, respectively, the displacement of the mass m along z assumes the following expression [43]:

(22) $$\Delta z=2\Omega_{x}\frac{F_{cz}}{m}\frac{Q_{y}}{\omega_{y}}\frac{1}{\sqrt{{\left(\omega^{2}{}_{y}+\omega^{2}{}_{z}\right)}^{2}+{\left(\frac{\omega_{y}\omega_{z}}{Q_{z}}\right)}^{2}}}$$

As expressed in Equation (22), the MEMS gyroscope sensitivity can be improved by matching the resonant frequencies *ω_y_* and* ω_z_*, and by reduction of friction (e.g., by creating an under vacuum operating environment) in order to improve the quality factor *Q_y/z_*.

Based on these physical principles, a brief panoramic of the research development of silicon MEMS gyroscope that were designed, prototyped and realized from the late 1980s to the 1990s. The following listed sensors are based on revealing Coriolis force and represent, someway, the milestone in the roadmap of MEMS gyro technology improvement.

The pioneering work to miniaturize inertial systems, made by Draper Laboratory (expert in inertial guidance systems for military and space applications), led to the creation of MEMS gyros and accelerometers as we know today. In the late 1980s, they developed a hand-assembled device to prove the feasibility of a silicon gyro. In the following work, they also successfully constructed a monolithic double gimbal gyro with a rate detection capability of 4°/s at a 1 Hz bandwidth; this performance was limited by noise [11].

Until 1996, in Draper Laboratory, other structures were designed to improve performance with an even simpler fabrication process. A planar design of a gyro based on the tuning fork principle was then presented. It was equipped with the comb drive mechanism developed at UC Berkeley, which showed a rate capability of about 0.1°/s in a 60 Hz bandwidth. Afterwards, they explored the fabrication advantages of the vibrating wheel on a gimbal-based gyro design capable of a better rate sensing [12,44].

Several researches, aimed to integrate part of control and signal processing on chip, to develop dual-axis gyroscope and to improve fabrication and performance with multi degree of freedom design, were made at UC Berkeley during next years.

In 1996, Clark, Howe, and Horowitz presented a *z*-axis vibratory rate gyroscope. This device integrated with a trans-resistance amplifier while the sense mode offers a differential measurement using interdigitated comb fingers. This device has a resolution of 1°/s/√Hz improvable to 0.1°/s/√Hz in a second generation.

After that, Juneau, Pisano and Smith reported a surface-micromachined dual-axis gyroscope based on a rotor disk that can equally sense rotation about two orthogonal axes. This device, integrated with some electronics, showed a random walk of 10°/√h and a cross-axis sensitivity comprised between 3% and 16% [45,46,47,48,49].

In 1999, Mochida, Tamura and Ohwada (Murata, Yokohama R&D Center) reported two designs of micromachined gyroscopes: a simple structure used as a reference, and another one with independent beams for drive and detection modes, thus uncoupling drive and sense mode for improving resolution. They characterized the two devices through laser meter equipment to observe displacements and the overall behavior, so they found that the gyroscope with independent beams had a resolution of 0.07°/s at a bandwidth of 10 Hz, even if limited by increased noise at resonance, caused by oscillation instability. This performance resulted to be better than that related to the reference device, due to weak coupling between the two modes [48].

Further development followed since the late 1990s, thanks to the fact that silicon technology became more mature, so it was possible to integrate control and processing electronic components into MEMS.

Studies aiming at improving MEMS gyroscopes increased, being already known the main aspects of theory and operation; the availability of more sophisticate test equipment to characterize prototypes, more powerful design tools and industrial interest in other application fields (at consumer level) contributed to the progress of this technology.

Georgia Institute of Technology also put great efforts in the research of MEMS gyroscopes. In this institution, the Matched-Mode Tuning Fork Gyroscope (M2-TFG) [50,51,52,53] was developed in 2006 by Zaman et al. It used an electrostatic comb drive to move the proof-masses in *x*-axis and a capacitive detecting in *y*-axis to sense rotation in *z*-axis. Drive and sense mode were electrostatically balanced to achieve perfect mode matching; this design improved sensitivity, bias stability and noise floor.

Sharma, through further research on the M2-TFG, designed the closed-loop circuit based on a transimpedance amplifier with a dynamic range of 104 dB, capable to keep the matched-mode. Experimental data showed a capacitive resolution of 0.02 aF/√Hz at 15 kHz. Zaman in 2008 reported an improvement of the M2-TFG using two high-quality factor resonant modes. The open-loop rate sensitivity of the new design was 83 mV/°/s in vacuum while the bias instability was 0.15°/h.

From 2009 to 2011, Trusov et al. at University of California developed at first the design of a *z*-axis MEMS gyroscope based on a tuning fork, then a new dual-mass vibratory MEMS *z*-axis rate gyroscope that improved the mechanical vibratory modes. These structures forced an anti-phase drive-mode and a linearly-coupled dynamically-balanced anti-phase sense-mode, that prioritizes sense-mode quality factor. The prototypes were characterized in a vacuum chamber, demonstrating a quality factor drive-mode of 67,000 and of 125,000 for the sense-mode [54,55].

Meanwhile, the joined forces of Old Dominion University and University of Utah led to the improvement of the M2-TFG architecture. In fact, Wang et al. presented a multiple beam tuning fork gyroscope that reached a measured Q-factor of 255,000 for drive-mode and 103,000 for sense-mode at 15.7 kHz. Further measurements pointed out a rate resolution of 0.37°/h/√Hz, a rate sensitivity of 80 VPP/°/s while ARW and bias instability were 6.67°/√h and 95°/h, respectively [56].

Other researches were performed towards enabling a wider bandwidth to expand flexibility and ease of use. Thus, in 2012, Tsai et al. developed a doubly decoupled MEMS gyroscope to minimize coupling between the drive-mode and sense-mode, which used frequencies in a 240 Hz bandwidth [57].

Finally, the report by Pyatishev et al. (2017) about a MEMS gyro characterized by a comb-shaped drive with enlarged capacity gradient considered the aspect ratio or the wavy aspect ratio of the comb drive as a fundamental key to improve performance [58].

## 5. Key Gyro Performance Factors

In this section, five critical parameters for consumer grade gyros will be overviewed:

(1) Angle Random Walk (ARW)
(2) Bias Offset Error
(3) Bias Instability
(4) Temperature Sensitivity
(5) Shock and Vibration Sensitivity

### 5.1. Angle Random Walk

In the output of a gyro, there is always a broadband white noise element. Angle Random Walk describes the error resulting from this noise element and can be evaluated using the Allan Variance technique. Active elements of the gyro are the major contributors to random noise (laser diode and photo diode for optical gyroscopes and the vibrating beam and detection electronics for MEMS). Noise is one of the most important differences between optical and MEMS gyro performance, resulting in different precision and accuracy in measurements.

### 5.2. Bias Offset Error

When input rotation is null, the output of the gyro could be nonzero. The equivalent input rotation detected is the Bias Offset Error. It is typically given at 25 °C for an ideal environment. Fixed errors, such as Bias Offset Error, can be easily corrected.

### 5.3. Bias Instability

Bias Instability is the instability of the bias offset at any constant temperature and ideal environment. It can be measured using the Allan Variance technique. Bias instability introduces errors that may not be easy to calibrate. Its influence is greater on longer measurement periods, so Bias Instability is one of the most critical factors in the gyro selection process for applications that requires excellent accuracy over long time.

### 5.4. Temperature Sensitivity

Gyro performance changes over temperature. A characterization of parameters such as noise, bias offset and scale factor over temperature is necessary to verify that gyro performance meets system targets.

### 5.5. Shock and Vibration Sensitivity

Noise and Bias offset of gyros also degrade under vibration and shock input. Vibration performance is critical in many military and industrial applications, because of the presence of numerous factors such as engines or gunfire.

### 5.6. Gyro Technology Comparison

The evolution of modern gyros technology, performance and application could be understood through an overview of its history starting from mid-19th century. Originally, it was a full mechanical system that found its major use in navy and aviation applications, especially during WWI as a pilot system for ship steering and for self-guided missiles. The first improvement step consisted in developing several DTG versions equipped with electronics. These kinds of systems are still used, even if their commercialization stopped some years after. In the 1970s, RLGs and FOGs emerged and became commercially mature in the next decade. Being very accurate but complex at the same time, large and expensive to manufacture, their main application consisted in replacing mechanical gyros components and systems in a wide variety of guidance, navigation and aeronautics applications, including man-portable and tripod target locator systems. In the last twenty years, a different technology, based on solid state integrated devices, appeared.

MEMS have achieved important improvements since first solutions to date. They have met the market request, in particular in consumer and industrial fields, allowing high robustness and sufficiently high performance for the corresponding grade. Nowadays the Car Braking System uses gyros in Electronic Stability Control. In consumer market, a large number of devices are provided with an embedded MEMS gyroscope. Segway-like Human Transporter, drones, smartphones and IOT devices are examples of markets and applications.

In industrial applications, the majority of systems where feedback control is needed, are equipped with a MEMS gyro, e.g., industrial automation units such as handling robots and factory transport systems.

FOG-grade MEMS gyros are currently in an advanced stage of development, paving the way to the replacement of optical gyros in the next future.

Actually, we can classify gyro technology considering the bias stability as fundamental performance parameter as shown in Table 1.

Fiber Optic Gyroscopes could be considered the low-cost version of Ring Laser Gyroscopes, being a mature technology with similar performance and sizes. Thus, development in fiber technology can lead to the design of high-performance FOGs. We may expect that RLG technology will be overtaken by FOG improvements and will be replaced by the last released FOGs. At the same time, a similar process will involve FOGs and MEMS gyro technologies, because they show a few significant advantages, such as reduction of size, power and cost, and it seems to be almost mature to move on the next performance grade. To date, the bias stability (around 5 to 30°/h) of MEMS cannot satisfy the tactical grade requirements, even if they have chance to overcome tactical RLGs and FOGs.

These trends are evident even in commercially available MEMS gyros and FOGs, such as those reported in [59,60,61,62]. In [59,60], two comparison tables on commercial MEMS gyros by two different manufacturers are shown. They focus on the most important performance parameters of their available products. The crucial point that influences the price of the product is the bias instability, which is one of the most important elements identifying the performance grade each gyro receives. All of the shown parameters (especially the dynamic range) are useful to choose the best gyro for the specific application.

In [61,62], comparative tables on commercial FOGs are shown. It is evident that the bias instability and the ARW of FOGs are much lower than those of MEMS. This is the reason why FOGs are more suitable for tactical grade applications, reaching more than the 50% of the market.

## 6. Companies Involved in the Development of Gyroscope Technologies

In this section, with reference to the previous gyroscope technologies, we report in Table 2 the companies, divided for geographic area, that actually are the main players in the gyroscope market. As can be seen in Table 2, the more diffused technologies of gyroscopes are actually the MEMS ones, followed by the RLGs. As discussed before, this market trend is related to the low cost of MEMS gyroscopes, allowing them to be employed for low-cost consumer electronics applications. North America is the geographic area where the gyroscope technologies are more developed and it is followed by Europe and Asia.

## 7. Conclusions

In this review, we reported the currently more diffused gyroscope technologies. The considered gyroscopes include mechanical gyroscopes and optical gyroscopes at macro- and micro-scale. In particular, commercially available gyroscope technologies, such as Mechanical Gyroscopes, silicon MEMS Gyroscopes, Ring Laser Gyroscopes (RLGs) and Fiber-Optic Gyroscopes (FOGs) are discussed, focusing attention on the main features, performances, technologies, applications and market players.

## Figures and Tables

**Table 1:** Gyro technology comparison in terms of Bias Stability.

Performance Grade	Bias Stability	Applications	Gyro Technology
Consumer	30–1000°/h	Motion interface	MEMS
Industrial & Low-end Tactical	1–30°/h	Ammunitions & rockets guidance	MEMS
Tactical	0.1–30°/h	Platform stabilization	FOG/RLG
High-end Tactical	0.1–1°/h	Missile navigation	RLG/FOG
Navigation	0.01–0.1°/h	Aeronautics navigation	RLG/FOG
Strategic	0.0001–0.01°/h	Submarine navigation	RLG/FOG

**Table 2:** Main players for gyroscope market.

Company	Gyroscope Technologies
**Asia**	
Aviation Gyro Photoelectricity Technology [63]	FOGS and RLGS
AVIC Xi’an Flight Automatic Control Research [64] Institute	MEMS gyroscopes, FOG and RLGs
MT Microsystems Co. Ltd. [65]	MEMS gyroscopes
Navtech inc. [66]	MEMS, FOG and laser gyroscopes
Panasonic Corporation [67]	Angular velocity sensors
Seiko Epson Corporation [68]	MEMS Gyroscopes and IMUs
**Europe**	
Northrop Grumman LITEF GmbH [69]	Gyroscopes, IMUs
Silicon Sensing Systems Ltd. [70]	Gyroscopes, IMUs
AIMS Sweden AB [71]	FOG gyroscopes
Civitanavi Systems s.r.l. [72]	Gyroscopes
InnaLabs Ltd [73]	Gyroscopes
Omni Instruments [74]	Gyroscopes, IMUs
Robert Bosch GmbH [75]	Gyroscopes,
Sensonor AS [76]	Gyroscopes, IMUs
STMicroelectronics N.V. [77]	Gyroscopes
Tronics’s Microsystems SA [78]	Gyroscopes
**North America**	
Analog Devices, Inc. [79]	MEMS gyroscopes s,
Emcore Corporation [80]	FOGs
Freescale Semiconductor, Inc. [81]	Gyroscopes
Gladiator Technologies, LKD Aerospace, Inc. [82]	MEMS gyroscopes, IMUs
Hewlett-Packard Development Company, L.P. [83]	MEMS Inertial sensors
Honeywell International Inc. [84]	RLGs,
InvenSense, Inc. [85]	MEMS gyroscopes, IMUs
Kearfott Corporation [86]	RLGs
Kionix Inc. [87]	Gyroscopes
KVH Industries, Inc. [88]	FOGs
LORD Corporation [89]	MEMS gyroscopes
Measurement Specialties, Inc. [90]	Gyroscopes,
Qualtre Inc. [91]	MEMS gyroscopes
Rockwell Collins Inc. [92]	Gyroscopes, IMU
Systron Donner Inertial [93]	MEMS gyroscopes
TE Connectivity Ltd. [94]	MEMS gyroscopes
Teledyne Technologies, Inc. [95]	RLGs
UTC Aerospace Systems [96]	Gyroscopes
Watson Industries Inc. [97]	MEMS Gyroscopes
**Russia**	
Fizoptika [98]	FOGs
Inertial Technologies JSC [99]	RLGs,
MIEA JSC [100]	RLGs,
OAO Polyus [101]	RLGs
Optolink Scientific Ltd. [102]	FOGs
Russian MEMS Association [103]	MEMS gyroscopes
**Other**	
Al Cielo Inertial Solutions Ltd. [104]	Gyroscopes, IMUs
Israel Aerospace Industries Ltd. [105]	Gyroscopes
